# Humanization helps build a harmonious doctor–patient relationship

**DOI:** 10.1590/1806-9282.20231497

**Published:** 2024-04-22

**Affiliations:** 1 Binzhou University, Department of Physical Education – Binzhou, China.; 2 Binzhou University, Department of Biological and Environmental Engineering – Binzhou, China.

In recent years, based on the frequent occurrence of medical disputes, the doctor–patient relationship has gradually become tense^1^, which not only seriously affects the medical industry but also becomes one of the social disharmony factors. A good doctor–patient relationship is a prerequisite for improving the compliance, satisfaction, and loyalty of patients and their families, and at the same time, doctors can reasonably take the best treatment measures to achieve satisfactory treatment results^2^. It shows that doctors have the key to establish a harmonious doctor–patient relationship.

Previously, in solving the doctor–patient relationship, through the formulation of various management policies and regulations, although it also played a partial effect, it is easy to cause over-reliance on the system of the rigid situation. As a matter of fact, the formulation of policies plays more of a constraining effect^3^ but cannot change the human mindset. Therefore, from the perspective of doctors’ social responsibility, the concept of humanized medical practice is the most effective way to solve the doctor–patient relationship.

Humanization is a comprehensive concept that encompasses several aspects. This study concludes that communication skills, service concepts, and medical ethics are the three most crucial aspects from a doctor's point of view. However, cultivating the concept of humanization should be implemented not only among professional doctors but also from the educational stage of medical students. The specifics are as follows.

1. Communication skills. Effective doctor–patient communication not only guides patients to accurately express their conditions and improves the accuracy of medical treatment but also establishes a trusting relationship between doctors and patients, reduces the degree of panic of patients about their diseases, and reflects more humanistic care.
2. Service consciousness. Doctors should adapt to the patient-centered diagnosis and treatment service consciousness, put the patient's needs and interests on the premise of diagnosis and treatment, give patients more humanistic care, so that patients get full attention and respect, and increase mutual trust.
3. Medical ethics education. Medical ethics is the behavioral principles and norms of those engaged in the medical profession. The education of medical ethics should be cultivated from the stage of medical students in school so that they can acquire the basic professional quality and establish correct values in advance.
